# THE “GOOD” CAREGIVER: DOES FILIAL PIETY NECESSITATE SELF-SACRIFICE?

**DOI:** 10.1093/geroni/igad104.1218

**Published:** 2023-12-21

**Authors:** Ad Maulod, Sasha Rouse, Nur ’Atiqah Lee, Rahul Malhotra

**Affiliations:** Duke-NUS Medical School, Singapore, Singapore; Duke-NUS Medical School, Singapore, Singapore; Duke-NUS Medical School, Singapore, Singapore; Duke-NUS Medical School, Singapore, Singapore

## Abstract

Family caregiving policies that amplify the enactment of ‘filial piety’ – respect and care towards parents, grandparents and older persons – are especially pronounced in Asian societies, where caring for older family members is culturally expected and appraised yet is associated with stress and burden. Numerous studies have shown that fulfilment of filial piety contributes to better psychological outcomes for caregivers while mismatched filial expectations contribute to poorer psychological outcomes. However, few studies acknowledge how filial care varies according to individual resources and socio-cultural contexts. We seek to understand how family caregivers negotiate their self-determination through filial care, which contributes to positive and negative caregiving experiences. We conducted in-depth interviews with 34 caregivers of older persons with functional limitations in Singapore, purposefully selected based on varying positive and negative caregiving experiences. For caregivers with predominantly negative caregiving experiences, filial care was seen as an act of sacrifice and expectation of duty, with low autonomy to exercise self-determination. In comparison, caregivers with predominantly positive caregiving experiences described filial care as an act of sincere love cultivated through close relationships with their care-recipient, and aligned to their self-determination. Strong family support was also evident in the latter. Treating filial piety as a ‘natural desire to care for parents’ obscures efforts required to amplify positive caregiving experiences while normalisation of self-sacrifice and suffering hinders caregivers from expressing burden and accessing support. A nuanced understanding of how caregivers negotiate filial care can inform policy interventions to empower caregivers and care-recipients and enhance intergenerational relationships.

